# Whole exome sequencing reveals a pathogenic homozygous CLDN16 mutation in a 17-year-old patient with familial hypomagnesemia with hypercalciuria and nephrocalcinosis: A case report

**DOI:** 10.1097/MD.0000000000043839

**Published:** 2025-08-15

**Authors:** Fei Wang, Yilinuer Adeerjiang, Hai-Qing Xing, Sheng Jiang

**Affiliations:** a Department of Endocrinology, The First Affiliated Hospital of Xinjiang Medical University, Urumqi, China; b State Key Laboratory of Pathogenesis, Prevention and Treatment of High Incidence Diseases in Central Asia, Urumqi, China; c College of Pharmacy, Macau University of Science and Technology, Macau Special Administrative Region, China.

**Keywords:** case report, CLDN16, hypercalciuria, hypomagnesemia, whole-exome sequencing

## Abstract

**Rationale:**

Familial hypomagnesemia with hypercalciuria and nephrocalcinosis (FHHNC) is a rare autosomal recessive renal tubular disorder caused by mutations in the CLDN16 or CLDN19 genes. This case demonstrates the critical role of whole-exome sequencing (WES) in diagnosing FHHNC, particularly when novel mutations, such as the homozygous CLDN16 variant (c.351G > T, p.W117C) found in this patient, are identified, which expands the genetic understanding of this condition.

**Patient concerns:**

A 17-year-old female presented with an 8-month history of persistent neck, shoulder, and upper limb pain that had worsened over the past month. Despite initial treatment for cervical degenerative disease at local hospitals, her symptoms showed minimal improvement. She also exhibited growth retardation, with a height of 145 cm (−2 SD), and experienced recurrent urinary tract infections, although she reported no visual disturbances or neurological symptoms.

**Diagnoses:**

Laboratory tests revealed hypomagnesemia and elevated parathyroid hormone levels. Imaging studies confirmed bilateral nephrocalcinosis and a medullary sponge kidney. Genetic testing using WES identified a homozygous pathogenic mutation in CLDN16 (c.351G > T), which was further validated by Sanger sequencing in the patient and her heterozygous parents.

**Interventions:**

During hospitalization, the patient received intravenous magnesium sulfate (2.5 g/day), oral calcium carbonate with vitamin D3 supplementation, and levofloxacin for urinary tract infection management. Upon discharge, she was maintained on oral magnesium oxide (500 mg twice daily) with regular monitoring of electrolyte levels and renal function.

**Outcomes:**

At the 2-year follow-up, the patient’s serum magnesium levels improved but remained below the normal range. Hypercalciuria persisted, although her renal function was stable. Notably, there was no progression to end-stage renal disease, and her symptoms were better managed with ongoing treatment.

**Lessons:**

This case underscores the importance of WES in the diagnosis of rare tubular disorders, especially when the clinical presentations are nonspecific. This finding highlights that CLDN16-related FHHNC can occur without ocular involvement, and this information may aid in differential diagnosis. Early and consistent magnesium supplementation appears to mitigate renal deterioration, emphasizing the need for prompt intervention. Additionally, this case reinforces the value of genetic counseling for families affected by autosomal recessive conditions.

## 1. Introduction

Familial hypomagnesemia with hypercalciuria and nephrocalcinosis (FHHNC) is a rare autosomal recessive renal tubular disorder characterized by a clinical triad of severe urinary magnesium (Mg) wasting, associated with hypercalciuria, nephrocalcinosis, and progression to kidney failure.^[1]^ Other clinical symptoms include seizures, tetany, failure to thrive, and polyuria with polydipsia.^[2]^

FHHNC is a monogenic disorder caused by loss-of-function mutations in the CLDN16 or CLDN19 genes encoding the tight junction proteins claudin-16^[3]^ and claudin-19,^[4]^ respectively. In the thick ascending limb (TAL) of the loop of Henle, claudin-16 and claudin-19 facilitate the paracellular reabsorption of Ca^2+^ and Mg^2+^. A proportion of ~25% and ~60%, respectively, of the freely filtered cations are reabsorbed in this part of the nephron.^[5]^ Patients with FHHNC suffer from defects in Ca^2+^ and Mg^2+^ reabsorption in the TAL with insufficient compensation in the distal convoluted tubules –the next nephron segment – where 5% to 10% of Ca^2+^ and Mg^2+^ are reabsorbed transcellularly via the Trpv5 and Trpm6 transporters.^[6]^ Whole-exome sequencing (WES) has been a useful tool for detecting the molecular pathology of this disease.

In this study, we report the use of WES to clarify the underlying genetic cause and reach a clinical diagnosis for a suspected case of FHHNC.

## 2. Case report

A 17-year-old girl presented to the Department of Endocrinology, The First Affiliated Hospital of Xinjiang Medical University, Urumqi, China, with neck, shoulder, and upper limb pain for 8 months, which worsened for 1 month in April 2020.


$$\begin{array}{l} {\text{H}eight:\;\;\;145\;\;\;cm,\;\;\;weight:\;\;\;43\;\;\;kg,\;\;\;BMI:\;\;\;20.45\;\;\;kg/m}^{\text{2}} \end{array}$$

The patient sought medical attention at a local hospital 8 months prior because of neck, shoulder, and upper limb pain. After thorough examination, the patient was diagnosed with cervical degenerative disease, and painkillers were given. However, the degree of pain relief was not significant. Afterward, she returned to the local hospital for treatment and was administered painkillers and nerve block therapy, but the effect was unsatisfactory. 1 month prior, the patient was admitted to our pain department for treatment. Blood metabolic profiling was performed. Blood biochemistry revealed increases in inorganic phosphorus, uric acid, alkaline phosphatase, osteocalcin and parathyroid hormone (PTH) levels and decreases in HCO3, calcium (Ca), Mg and 25-hydroxyvitamin D levels (Table 1). The patient was transferred to the endocrinology department for further diagnostic clarification.

**Table 1:** Blood profiling results in the pain department.

Blood	Result	Unit	Normal
Potassium (K)	3.78	mmol/L	3.5–5.3
Sodium (Na)	142.00	mmol/L	137–147
Chloride (Cl)	109.2	mmol/L	99–110
HCO3	21.70 ↓	mmol/L	23–29
Calcium (Ca)	1.69 ↓	mmol/L	2.11–2.52
Magnesium (Mg)	0.51 ↓	mmol/L	0.75–1.02
Inorganic phosphorus	1.92 ↑	mmol/L	0.85–1.51
Uric acid	439.70 ↑	umol/L	155–357
Creatinine (Cr)	87.32	umol/L	53–115
Glomerular filtration rate (eGFR)	84.27		56–122
Alkaline phosphatase (AKP)	158.60 ↑	U/L	35–100
25-Hydroxyvitamin D	24.05 ↓	nmol/L	>50
Osteocalcin (OC)	88.49 ↑	ng/mL	13–48
Parathyroid hormone (PTH)	18.30 ↑	pmol/L	1.6–6.9

Blood metabolic profiling and urine analysis were performed. Blood biochemistry revealed increases in inorganic phosphorus, alkaline phosphatase, total type I collagen amino terminal elongation peptide, β-collagen-specific sequence and PTH levels and decreases in Ca and Mg levels. Urine analysis also revealed abnormal results, including occult blood 3+, leukocyte 3+, red blood cell count 480/µL, white blood cell count 221/µL, bacterial count 10/µL, and low 24-hour potassium, 24-hour Ca and 24-hour chloride levels (Table 2). General bacterial culture + drug sensitivity test (urine): *Escherichia coli* with a bacterial count of > 10^4^ CFU/mL.

**Table 2:** Blood and urine profiling results from the endocrinology department.

Blood	Result	Unit	Normal
Calcium (Ca)	1.69 ↓	mmol/L	2.11–2.52
Magnesium (Mg)	0.46 ↓	mmol/L	0.75–1.02
Inorganic phosphorus	2.21 ↑	mmol/L	0.85–1.51
Creatinine (Cr)	99.5	umol/L	53–115
Glomerular filtration rate (eGFR)	71.96		56–122
Alkaline phosphatase (AKP)	146.2 ↑	U/L	35–100
Total type I collagen amino terminal elongation peptide	219 ↑	ng/mL	15.13–59.59
β-Collagen specific sequence	2.48 ↑	ng/mL	0.025–0.573
Parathyroid hormone (PTH)	19.05 ↑	pmol/L	1.6–6.9
Urine	Result	Unit	Normal
Occult blood	3+		–
Leukocyte	3+		–
Red blood cell count	489	/uL	0–17
White blood cell count	221	/uL	0–28
Bacterial count	10	/uL	0–7
24 h random potassium	11.7 ↓	mmol/24 h	25–100
24 h random calcium	63.24 ↓	mmol/24 h	130–260
24 h random chloride	51.24 ↓	mmol/24 h	170–250
24 h random calcium	3.9	mmo/24 h	2.5–7.5
PH	5.5		5–8

**Figure 1.**
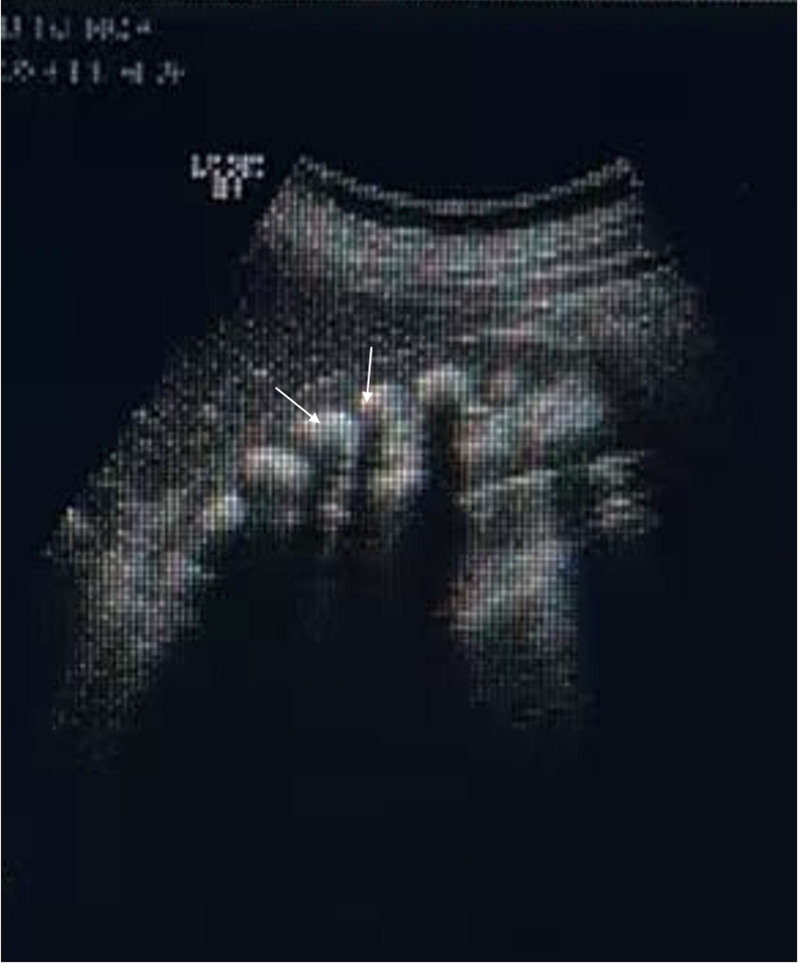
Sonography of kidneys. Strong echoes can be seen in diffuse pyramids of both kidneys, with larger ones measuring approximately 1.6 cm and accompanied by acoustic shadows.

CT: multiple symmetrically distributed positive stones in both renal parenchyma and sponges in the kidney were revealed. Ultrasound: diffuse intravertebral stones in both kidneys and a medullary sponge kidney were revealed (Fig. 1). Pelvic radiograph: there was no significant change in the density of the pelvic bone structure morphology. X-ray of the left and right femurs: no clear abnormalities were found in the morphology or density of the bone structure of both femurs. Bone age film. In the bone film, 8 wrist bones were observed in the left wrist, and the epiphyseal lines in the left hand and wrist were closed.

**Figure 2.**
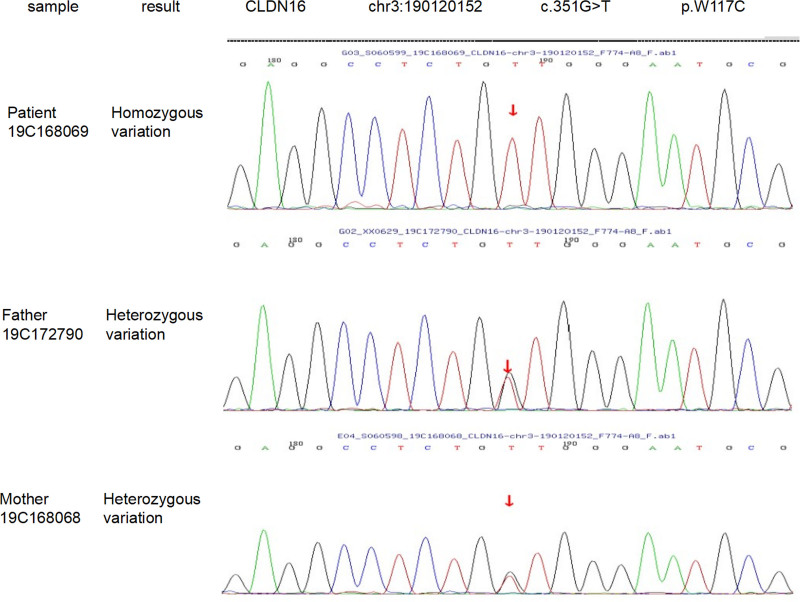
Sanger sequencing in the family trio. The arrows indicate the position affected by the mutation in the patient and her parents.

WES was performed on the peripheral blood sample of the patient, revealing a homozygous variant in exon 2 of the CLDN16 gene, specifically c.351G > T (p.W117C). To further validate the inheritance pattern of this variant, Sanger sequencing was conducted on the peripheral blood samples obtained from the patient’s parents. The results revealed that the father has a heterozygous variant in the second exon (c.351G > T, p.W117C), and the mother also has a heterozygous variant in the second exon (c.351G > T, p.W117C) (Fig. 2).

On the basis of the testing results, the patient carries a homozygous variant c.351G > T (p.W117C), whereas both parents are heterozygous carriers of the same variant. This pattern is consistent with autosomal recessive inheritance, indicating that the patient inherited 1 mutant allele from each parent, resulting in a homozygous mutation.

During hospitalization, the patient received Ca supplementation with calcium carbonate and vitamin D3 tablets (1 tablet twice daily orally), vitamin D drops (400 IU twice daily orally), Mg supplementation including 25% magnesium sulfate injection (10 mL/2.5 g once daily intravenously) and potassium magnesium aspartate tablets (2 tablets 3 times daily orally), along with anti-infective treatment using levofloxacin tablets (0.5 g once daily orally). Following discharge, the treatment regimen was adjusted to oral magnesium oxide maintenance therapy (1 tablet twice daily orally).

The requirement for approval of this case report was waived by the ethics committee at The First Affiliated Hospital of Xinjiang Medical University owing to the retrospective nature of the study. All procedures in this study were performed in accordance with the ethical standards of the institution and with the Helsinki Declaration (as revised in 2013). Written informed consent for the publication of this case report and accompanying images was not obtained from the patient or the relatives after all possible attempts were made.

The patients’ clinical and genetic data were collected as part of routine diagnostic and therapeutic procedures. All the data used in this study were anonymized to ensure patient privacy and confidentiality. No identifiable information, including names, initials, hospital identification numbers, or any other personal details, has been included in the manuscript or supplementary materials. The publication of this case report does not compromise the privacy or confidentiality of the patient in any way.

## 3. Discussion

Rodriguez-Soriano et al^[7]^ found that the pathogenesis of FHHNC involves impaired reabsorption of Ca and Mg in the TAL of the renal tubule. Simon et al^[3]^ identified the pathogenic gene PCLN1 for FHHNC, which is located on chromosome 3q27 and is currently known as CLDN16. Konrad et al^[4]^ identified the second pathogenic gene for FHHNC, CLND19, which is located on chromosome 1p34.2. Currently, 56 pathogenic mutations of the CLDN16 gene have been reported, primarily missense mutations, nonsense mutations, splice site mutations, and fragment mutations. These pathogenic mutations can be homozygous or compound heterozygous.^[8]^ The case presented here is a homozygous mutation, with parents carrying heterozygous mutations, and the homozygous mutation is pathogenic. This information suggests that the patient inherited the variant from both parents, resulting in a homozygous mutation, whereas each parent carried a heterozygous mutation in the same gene. The pathogenic mutation of the CLDN16 gene, c.351G > T, p.W117C, is preliminarily categorized as of uncertain clinical significance (uncertain) according to the ACMG guidelines. This classification is based on PM1 + PM2 + PP3. Specifically, PM1 indicates that the mutation is located in a hotspot region, PM2 shows a low frequency variant (-) in the normal population database, and PP3 reveals a harmful effect by the comprehensive bioinformatics protein function predictor REVEL, with SIFT, PolPhen_2, MutationTaster, and GERP + all predicting harmful outcomes. Familial verification analysis revealed that the father of the tested individual has a heterozygous variant at this site, and the mother also has a heterozygous variant. In addition, the tested individual has a homozygous variant at the same site. The parents are unaffected, whereas the tested individual is affected. This finding is consistent with Mendelian inheritance. This case presents a homozygous mutation of the CLDN16 gene leading to FHHNC. Claudin-16 is expressed only in the kidney, whereas Claudin-19 is also expressed in the retina and peripheral neurons.^[9]^ The disease was considered renal hypomagnesemia type 3 (OMIM: 248250), AR. Renal hypomagnesemia type 3 is a progressive kidney disease that typically presents in adolescence with renal Mg wasting, hypermagnesiuria, hypomagnesemia, hypercalcemia, and renal calcifications. Some patients may also experience recurrent urinary tract infections (UTI) and kidney stones. The clinical presentation of the patient in this case included slightly low blood Ca, no hypercalciuria, and low Mg levels. Owing to testing equipment constraints, 24-hour urinary Mg was not monitored. The patient had a UTI combined with kidney stones and exhibited growth and development delays, with height and weight lagging behind those of peers (−2 SD). Hyperparathyroidism is common in all patients with renal calcification; thus, serum PTH levels are elevated in FHHNC patients.^[10]^ Elevated PTH levels are related to hypocalcemia and low vitamin D levels, with no evidence of osteoporosis. The disease was considered renal hypomagnesemia type 3 (OMIM: 248250), AR. Renal hypomagnesemia type 3 is a progressive kidney disease that typically presents in adolescence with renal Mg wasting, hypermagnesiuria, hypomagnesemia, hypercalcemia, and renal calcifications. Some patients may also experience recurrent UTIs and kidney stones. The clinical presentation of the patient in this case included slightly low blood Ca, no hypercalciuria, and low Mg levels. Owing to testing equipment constraints, 24-hour urinary Mg was not monitored. The patient had a UTI combined with kidney stones and exhibited growth and development delays, with height and weight lagging behind those of peers (−2 SD). Hyperparathyroidism is common in all patients with renal calcification; thus, serum PTH levels are elevated in FHHNC patients.^[11]^ Another notable feature of FHHNC is its progression to end-stage renal disease (ESRD). The uricosuric defects commonly observed in FHHNC are associated with distal renal tubular acidosis (mostly incomplete), along with defects in ammonia transport to the distal nephron.^[12]^ The hyperuricemia in FHHNC cannot be fully explained by a declining GFR. Patients with CLDN16 mutations may also exhibit mild ocular abnormalities. However, this patient showed no visual changes, astigmatism, strabismus, hyperopia, myopia, or nystagmus. Moreover, CLDN19 mutations may manifest with neurological symptoms, primarily exercise intolerance, weakness, and spasms, all of which were noted in this case.

To confirm the diagnosis of FHHNC, the triad of clinical symptoms, namely, hypomagnesemia, hypercalciuria, and renal calcifications, must be present, followed by genetic testing to identify pathogenic mutations in the CLDN16 or CLDN19 genes. In some patients, hypomagnesemia may not be pronounced due to a decline in the glomerular filtration rate, and at this point, a calculation of the urine Mg excretion fraction is necessary. FHHNC is a rare genetic disorder that is not easily diagnosed clinically. If pediatric or adolescent patients present with renal calcifications and have no significant history of other organ diseases or medication usage, FHHNC should be suspected. Genetic testing is mainly used to confirm clinical/biochemical/imaging diagnoses, assess familial genetic risk, and guide genetic counseling.

There is no specific treatment for this disease; current treatment is mainly supportive, which includes oral supplementation of Mg^2+^ and citrates, as well as thiazide diuretics to lower urinary Ca levels and control the progression of kidney calcifications.^[13]^ Conversely, thiazide diuretics may exacerbate low citrate levels and hyperuricemia. In this case, the patient had elevated uric acid and did not receive thiazide diuretic treatment.^[14]^ Renin-angiotensin system inhibitors may be employed to slow CKD progression.^[15]^ Despite controversies surrounding the implementation of vitamin D supplementation, given the risk of increased intestinal Ca absorption and worsening hypercalciuria, vitamin D is also expected to lower PTH levels, thereby reducing hypercalciuria. In this case, vitamin D3 drops were used, resulting in decreased PTH levels. Additionally, evidence suggests that normal vitamin D levels are correlated with a reduction in CKD progression.^[16]^ Research indicates that the risk of patients with FHHNC caused by CLDN19 mutations progressing to ESRD is twice that of patients with FHHNC caused by CLDN16 mutations.^[17]^ If one of the 2 allele mutations causing the disease from the CLDN16 gene retains partial function, the onset age and renal function impairment may be delayed.^[9]^ However, there are also reports showing significant clinical phenotype differences among different patients with the same pathogenic mutation in the same family.^[18]^ Once patients progress to ESRD, they require kidney transplantation. Following kidney transplantation, patients’ blood Mg and urinary Ca can return to normal, and the disease does not recur.^[13]^

After 2 years of follow-up, the patient adhered to treatment and was taking magnesium oxide, which caused some gastrointestinal discomfort but was tolerable. Reassessment of serum electrolytes revealed elevated blood Mg (not reaching the normal range), elevated blood Ca, normal phosphate, and normal urinary Ca levels but high urinary Mg levels. Renal function reassessment revealed normal serum creatinine levels but a low glomerular filtration rate. The patient is advised to have long-term follow-up, monitoring changes in blood Ca, blood Mg, urinary Ca, and urinary Mg levels as well as kidney function.

## Author contributions

**Conceptualization:** Sheng Jiang.

**Supervision:** Sheng Jiang.

**Writing – original draft:** Yilinuer Adeerjiang.

**Writing – review & editing:** Fei Wang, Yilinuer Adeerjiang, Hai-Qing Xing.
